# A Chromosomally Encoded Virulence Factor Protects the Lyme Disease Pathogen against Host-Adaptive Immunity

**DOI:** 10.1371/journal.ppat.1000326

**Published:** 2009-03-06

**Authors:** Xiuli Yang, Adam S. Coleman, Juan Anguita, Utpal Pal

**Affiliations:** 1 Department of Veterinary Medicine, University of Maryland, College Park, Maryland, United States of America; 2 Virginia–Maryland Regional College of Veterinary Medicine, College Park, Maryland, United States of America; 3 Department of Veterinary and Animal Sciences, University of Massachusetts at Amherst, Amherst, Massachusetts, United States of America; Medical College of Wisconsin, United States of America

## Abstract

*Borrelia burgdorferi*, the bacterial pathogen of Lyme borreliosis, differentially expresses select genes *in vivo*, likely contributing to microbial persistence and disease. Expression analysis of spirochete genes encoding potential membrane proteins showed that surface-located membrane protein 1 (*lmp1*) transcripts were expressed at high levels in the infected murine heart, especially during early stages of infection. Mice and humans with diagnosed Lyme borreliosis also developed antibodies against Lmp1. Deletion of *lmp1* severely impaired the pathogen's ability to persist in diverse murine tissues including the heart, and to induce disease, which was restored upon chromosomal complementation of the mutant with the *lmp1* gene. Lmp1 performs an immune-related rather than a metabolic function, as its deletion did not affect microbial persistence in immunodeficient mice, but significantly decreased spirochete resistance to the borreliacidal effects of anti-*B. burgdorferi* sera in a complement-independent manner. These data demonstrate the existence of a virulence factor that helps the pathogen evade host-acquired immune defense and establish persistent infection in mammals.

## Author Summary

The pathogen of Lyme borreliosis, *Borrelia burgdorferi*, causes disease in many parts of the world, resulting in multi-system complications in infected humans and animals. The microbe produces certain antigens in response to host environments that potentially allow it to persist and cause disease. Here, we analyzed the expression of *B. burgdorferi* genes encoding potential membrane proteins in infected hosts and show that one of them, termed Lmp1, is dramatically expressed in infected mice, most prominently in cardiac tissue during early infection. Mice and humans diagnosed with Lyme borreliosis also develop antibodies against Lmp1. Deletion of *lmp1* in an infectious isolate of *B. burgdorferi* impairs the pathogen's ability to persist in murine tissues, especially the heart, and to induce disease, which was reversed when the gene was inserted back into the chromosome of the mutant. Lmp1 performs an immune-related, rather than a metabolic, function as its deletion does not affect microbial persistence in immunodeficient mice, but decreases the spirochete's ability to resist the borreliacidal effects of anti-*B. burgdorferi* sera. These data identify the existence of a surface-located antigen of *B. burgdorferi* that helps the pathogen evade host-acquired immune defense and establish persistent infection and disease in mammals.

## Introduction

Lyme borreliosis, caused by *Borrelia burgdorferi* sensu lato, is the most prevalent tick-borne human disease in the United States, Europe and many parts of Asia [1]. Once the pathogen is deposited in the mammalian dermis by feeding *Ixodes* ticks, it establishes a localized infection at the bite site, then disseminates to distant cutaneous sites and various internal organs, including the spleen, bladder, joints, heart and central nervous system [1]–[3]. While *B. burgdorferi* persists in several tissue locations in mammals, only a limited set of organs, most frequently the joints and the heart, experience robust host-inflammatory responses resulting in clinical complications, such as Lyme arthritis and carditis. Antibiotic treatment is usually, but not always, successful, and some patients develop a form of antibiotic-resistant arthritis that is thought to be unrelated to persistent infection [4].

The *B. burgdorferi* transcriptome undergoes dynamic changes during the complex enzootic cycle of the spirochetes [5]–[8]. *B. burgdorferi* grown in laboratory medium or within host-implanted dialysis membrane chambers readily responds to altered environments, adapting to changes in temperature, pH, nutrients, and host immune responses [6], [9]–[15]. A significant fraction of the *B. burgdorferi* genome (8.6%), or 150 genes, could be differentially expressed *in vitro* in response to physiochemical alterations in growth conditions, and a major proportion of these genes (46%) encode proteins with predicted export signals [13]. However, while all *B. burgdorferi* lipoproteins have outer membrane export signals, some are retained in the periplasm by sequence-specific signals [16]. Studies have identified a few *B. burgdorferi* genes which are preferentially expressed in specific mammalian and arthropod environments and gene deletion studies [17] have confirmed that some of those differentially-expressed gene products support spirochete infectivity. For example, the *B. burgdorferi* genes *bbk32*, *dbpA/B* and *bmpA/B* are selectively expressed in mammals and facilitate *B. burgdorferi* infection of the murine host [8],[18],[19]. In contrast, *ospA*/*B, bb0365* and *bb0690* are highly expressed during specific stages of *B. burgdorferi* persistence in ticks and support the spirochete life cycle in the arthropod [7],[20],[21]. Other genes, such as *ospD* is dispensable for infectivity [22],[23], regardless of tightly regulated expression *in vivo* [22]. As gene duplication is a crucial mechanism of evolutionary innovation and the *B. burgdorferi* genome harbors significant clusters of paralogous genes in addition to large numbers of unique genes with unknown functional annotations [24],[25], many spirochete proteins may have functional redundancy. Thus, despite selective expression and beneficial contribution to the spirochete life cycle, antigens could be functionally redundant and non-essential for infectivity. Therefore, further identification of virulence genes that have significant impact on *B. burgdorferi* survival *in vivo* and pathogenesis is important for the development of preventative strategies.

The clinical complications of Lyme borreliosis are primarily triggered by *B. burgdorferi*-induced host inflammatory responses [26]–[28]. Although spirochetes colonize a wide variety of host tissues, the inflammatory response that results in pathology is observed in a limited set of host organs, most commonly in one or both mouse ankles or human knees and the heart. The diversity of host niches likely influences spirochete gene expression. While microbial antigens that are expressed at higher levels in a time- or tissue-specific manner may assist in *B. burgdorferi* persistence in local environments, antigens, especially those exposed on the microbial surface, could directly participate in host–pathogen interactions contributing to the genesis of organ-specific pathogenesis. Therefore, we assessed the expression levels of a selected set of *B. burgdorferi* genes in diverse murine tissues because of their putative membrane localization. We sought to determine if *B. burgdorferi* gene products that are preferentially expressed at high levels in clinically-relevant host microenvironments directly contribute to microbial virulence. The characterization of microbial ligands that are differentially expressed during the pathogen's life cycle is important for the identification of novel vaccine targets and the prevention of the multi-system disorders caused by *B. burgdorferi*.

## Results

### Identification of *B. burgdorferi* genes that are expressed in higher levels in infected murine tissues

*B. burgdorferi* persists in diverse tissue environments of the mammalian host. To identify *B. burgdorferi* genes that are expressed at high levels *in vivo*, particularly in a tissue-specific manner, we employed a sensitive quantitative RT-PCR (qRT-PCR) approach to compare spirochete transcriptomes in multiple murine tissues and *in vitro*. A total of 91 spirochete genes were selected for expression analysis, based on their putative association with the spirochete membrane as determined by the database annotation and *in silico* analysis for extracellular exposure (Table S1). Groups of C3H/HeN mice (5 animals/group) were challenged with *B. burgdorferi* (10^5^ cells/mouse) and skin, joints, heart and bladder tissue were collected following 1, 2, 3 and 4 weeks of infection. Total RNA was isolated, and corresponding tissues from the indicated time points were combined into four separate pools of skin, joint, heart and bladder samples. qRT-PCR analysis was performed using gene-specific primers as detailed in the Materials and Methods section. Analysis of qRT-PCR data revealed that 44 *B. burgdorferi* genes (out of 91 assessed) were not transcribed at detectable levels *in vivo*. The remaining 47 genes displayed variable expression across different tissues, which is presented as fold increase in transcript levels relative to *flaB*, together with corresponding *in vitro* expression levels (Figure S1). *B. burgdorferi bb0210*, annotated as surface-located membrane protein 1 (*lmp1*), which encodes an exported protein with type I signal peptide with unknown function [25], displayed the most dramatic differential expression in murine tissues, with the highest level of expression found in the heart (Figure S1).

### *lmp1* is dramatically expressed in the murine heart during early *B. burgdorferi* infection

**Figure 1:**
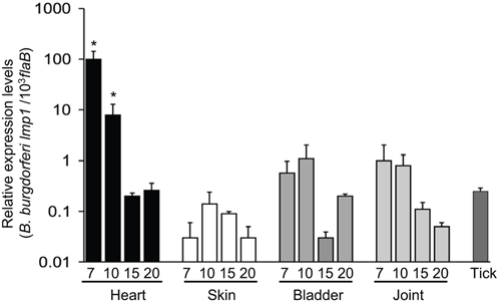
*B. burgdorferi lmp1* is dramatically upregulated during early phases of spirochete infection of the murine heart. Total RNA was isolated from murine heart, skin, bladder, and joints of *B. burgdorferi*–infected mice (5 mice/group) at days 7, 10, 15, and 20, following spirochete challenge, as well as from engorged nymphs that fed on 2-week–infected mice, which were then converted to cDNA for measuring *lmp1* transcripts using quantitative PCR. The relative expression levels of *lmp1* are presented as copies of *lmp1* transcript per 1,000 copies of *flaB* transcripts. Bars represent the mean±SEM from quantitative PCR analyses of three independent infection experiments. The transcript levels of *lmp1* in the heart on days 7 and 10 were significantly higher than all time points for the skin, joint, bladder, and tick (*P<0.01–0.001).

We used the initial qRT-PCR screen as a guide to focus on genes of potential importance, and performed more detailed temporal and spatial expression analyses of *lmp1* throughout the *B. burgdorferi* infection in mice. A similar qRT-PCR experiment (Figure S1), using separate RNA samples collected at weekly intervals, showed that *lmp1* was expressed at high levels between weeks 1 and 2, but produced at low levels between weeks 3 and 4 (data not shown). Therefore, a detailed expression analysis of *lmp1* focused on the early phases of *B. burgdorferi* infection in the murine host. To accomplish this, groups of C3H/HeN mice (5 animals/group) were infected with *B. burgdorferi*, and tissues were isolated at 7, 10, 15, and 20 days. Isolated total RNA was converted to cDNA and subjected to qRT-PCR to measure copies of *lmp1* transcripts, relative to *flaB* expression. The expression of *lmp1* was selectively upregulated in the heart at 7 and 10 days post-infection, compared to that in the skin, joints, bladder and infected ticks (Figure 1). Similar to syringe-based infection, the expression of *lmp1* was also significantly higher in infected hearts compared to other tissues, when mice were infected via natural tick-borne *B. burgdorferi* infection. The expression of *lmp1* in the murine heart, analyzed at day 7 following *B. burgdorferi*–infected tick challenge, was significantly higher than corresponding *lmp1* expression levels in the infected skin, bladder and joints (Figure S2). Consistent with *lmp1* expression data, mice infected with *B. burgdorferi* and human patients with diagnosed Lyme disease also developed detectable antibody responses to Lmp1 (Figure S3). These results suggest that *lmp1* encodes an immunogenic antigen that is expressed *in vivo*, with dramatic expression in the murine heart in both syringe and tick-transmitted *B. burgdorferi* infection. Therefore, we further examined the role of Lmp1 in *B. burgdorferi* virulence in mice.

### *lmp1* deletion interferes with the ability of the mutant *B. burgdorferi* to persist in murine tissues and to induce disease

**Figure 2:**
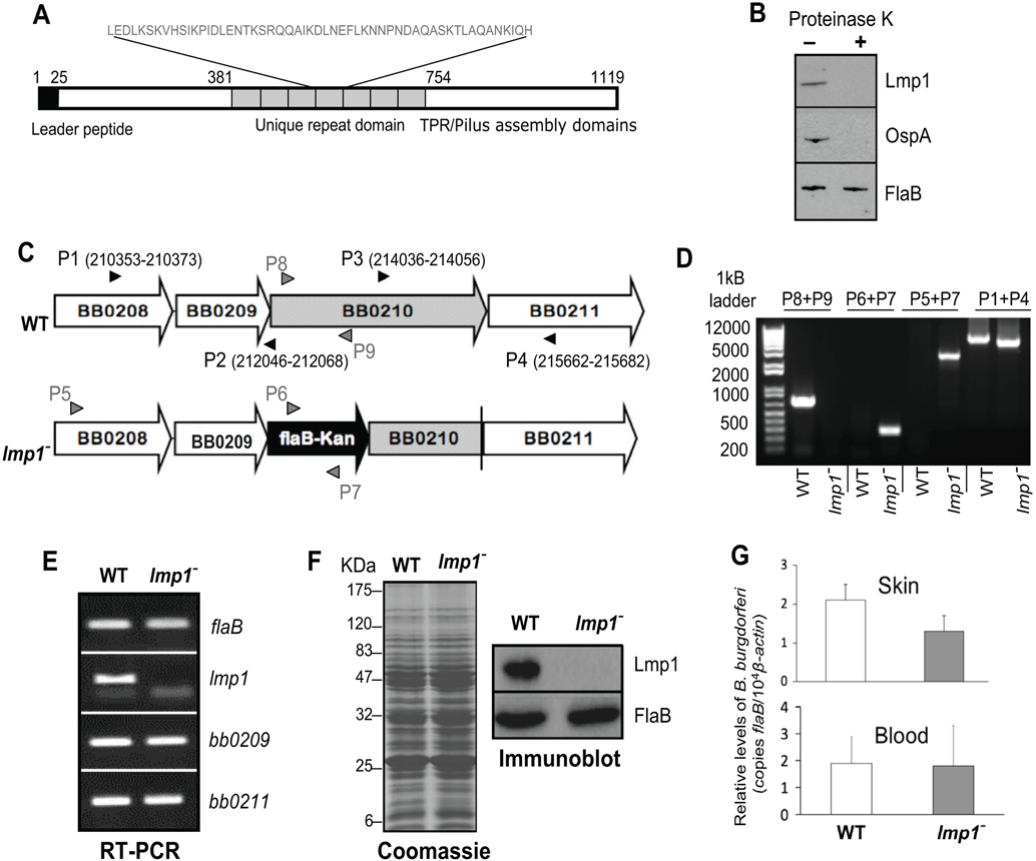
Construction and analysis of *lmp1* mutant *B. burgdorferi*. (A) Schematic diagram representing putative domain features of the Lmp1 protein. Amino acid positions at the beginning of the putative domains are indicated on the top. The leader peptide cleavage site predicted by SignalP 3.0 [58] is located after the 25^th^ amino acid position. The central region of the protein harbors seven unique repeat domains of 54 amino acids (indicated in gray letters), previously identified to house a potential *B. burgdorferi* adhesin motif [34]. The carboxyl terminal region contains putative conserved domains similar to tetratricopeptide repeat (TPR) or a pilus assembly protein, as identified by NCBI BLASTP conserved domains search program. Lmp1 sequence can be accessed via JCVI *B. burgdorferi* genome page with the locus tag BB_0210 or via GenBank accession number AAC66595.1 (B) Lmp1 is sensitive to proteinase K–mediated degradation of *B. burgdorferi* surface proteins. Viable spirochetes were incubated with (+) or without (−) proteinase K for the removal of protease-sensitive surface proteins and processed for immunoblot analysis using Lmp1 antibodies. (B) *burgdorferi* OspA and FlaB antibodies were utilized as controls for surface-exposed and sub-surface proteins, respectively. (C) Schematic drawings of the wild type isolate (WT) and the *lmp1* mutant *B. burgdorferi* (*lmp1^−^*) at the *bb0210* (*lmp1*) locus (gray box arrow). Genes *bb0208*, *bb0209*, and *bb0211* (white box-arrows) and the kanamycin-resistance cassette driven by the *B. burgdorferi flaB* promoter (flaB-Kan, black box-arrow) are indicated. Nucleotide positions of primers P1–P4 in the *B. burgdorferi* genomic database (www.tigr.org) are indicated. Seven nucleotides' overlap between the end of *bb0209* and the start of *bb0210* was not deleted in the allelic exchange by extending the primer P2 until the end of *bb0209*. The 5′ and the 3′ arms for homologous recombination flanking upstream and downstream of the *lmp1* locus were amplified using primers P1–P2 and P3–P4 (black arrowheads), and ligated to the flaB-Kan cassette as detailed in Materials and Methods. (D) Integration of the mutagenic construct, flaB-Kan, in the intended genomic locus. Primers 5–9 (gray arrowheads, positions indicated in Figure 2C) were used for PCR analysis with isolated DNA from wild type or *lmp1* mutant *B. burgdorferi* as templates. (E) RT-PCR analysis of wild type or *lmp1* mutant *B. burgdorferi*. Total RNA was isolated from cultured spirochete, and transcript levels of *lmp1*, *flaB*, *bb0209*, and *bb0211* were detected by RT-PCR analysis. (F) The lysates of wild type and *lmp1* mutant *B. burgdorferi* were separated on a SDS-PAGE gel and either stained with Coomassie blue (left) or transferred onto a nitrocellulose membrane and probed with the antiserum against Lmp1 or FlaB (right). Migration of protein standards is shown to the left in kDa. (G) The *lmp1* mutant *B. burgdorferi* were capable of dissemination from the dermis of infected mice. Skin and blood samples were collected from mice seven days after challenge, and the *B. burgdorferi* load was analyzed by quantitative PCR measurement of *flaB* copies and presented as *flaB*/murine *β-actin*.

Although the function of *B. burgdorferi* Lmp1 is unknown, the protein displays putative conserved domains including unique repeat modules housed in the central portion of the protein (Figure 2A). Polyclonal antibodies that specifically recognized native *B. burgdorferi* Lmp1 were generated (Figure S4) and used in a proteinase K accessibility assay, which indicated that Lmp1 is exposed on the microbial surface (Figure 2B). To further study the role of the Lmp1 in *B. burgdorferi* infectivity, we created *lmp1*-deficient *B. burgdorferi*. An isogenic mutant of *lmp1* was created by replacing part of the *lmp1* open reading frame with a kanamycin resistance cassette via homologous recombination (Figure 2C). A DNA construct was generated for the intended recombination, sequenced to confirm identity and transformed into *B. burgdorferi* as detailed in the Materials and Methods section. Transformants were further screened using PCR analysis to ensure that the antibiotic cassette was inserted into the intended chromosomal locus (Figure 2D), and that the plasmid profiles of the wild type and mutant spirochetes were identical (data not shown). RT-PCR analysis showed that while *lmp1* mRNA was undetectable in the *lmp1* mutant, similar to the parental isolate, the mutant was able to transcribe the surrounding genes, *bb0209* and *bb0211* (Figure 2E). qRT-PCR analysis further confirmed that the transcript levels of *bb0209* and *bb0211* in the *lmp1* mutant were 95(±9%) and 85 (±8%) of the respective wild type levels (data not shown). The *lmp1* mutant spirochetes contained a similar protein profile to that of the wild type (Figure 2F, left panel) and, as expected, the *lmp1* mutant did not produce Lmp1 protein (Figure 2F, right panel). We next compared the murine infectivity of *lmp1* mutant *B. burgdorferi* with that of the parental isolates. Groups of 10 C3H/HeN mice were inoculated intradermally with equal numbers of wild type or *lmp1* mutant *B. burgdorferi* (10^5^ spirochetes/mouse). qRT-PCR analysis (Figure 2G) and culture (data not shown) of murine skin biopsy and blood samples collected following one week of infection indicated that both *lmp1* mutants and wild type spirochetes were readily detectable in skin and blood. When *Ixodes* ticks were allowed to feed on mice inoculated with *lmp1*-deficient *B. burgdorferi* after two-weeks of infection, the mutants were able to migrate into feeding ticks (data not shown).

**Figure 3:**
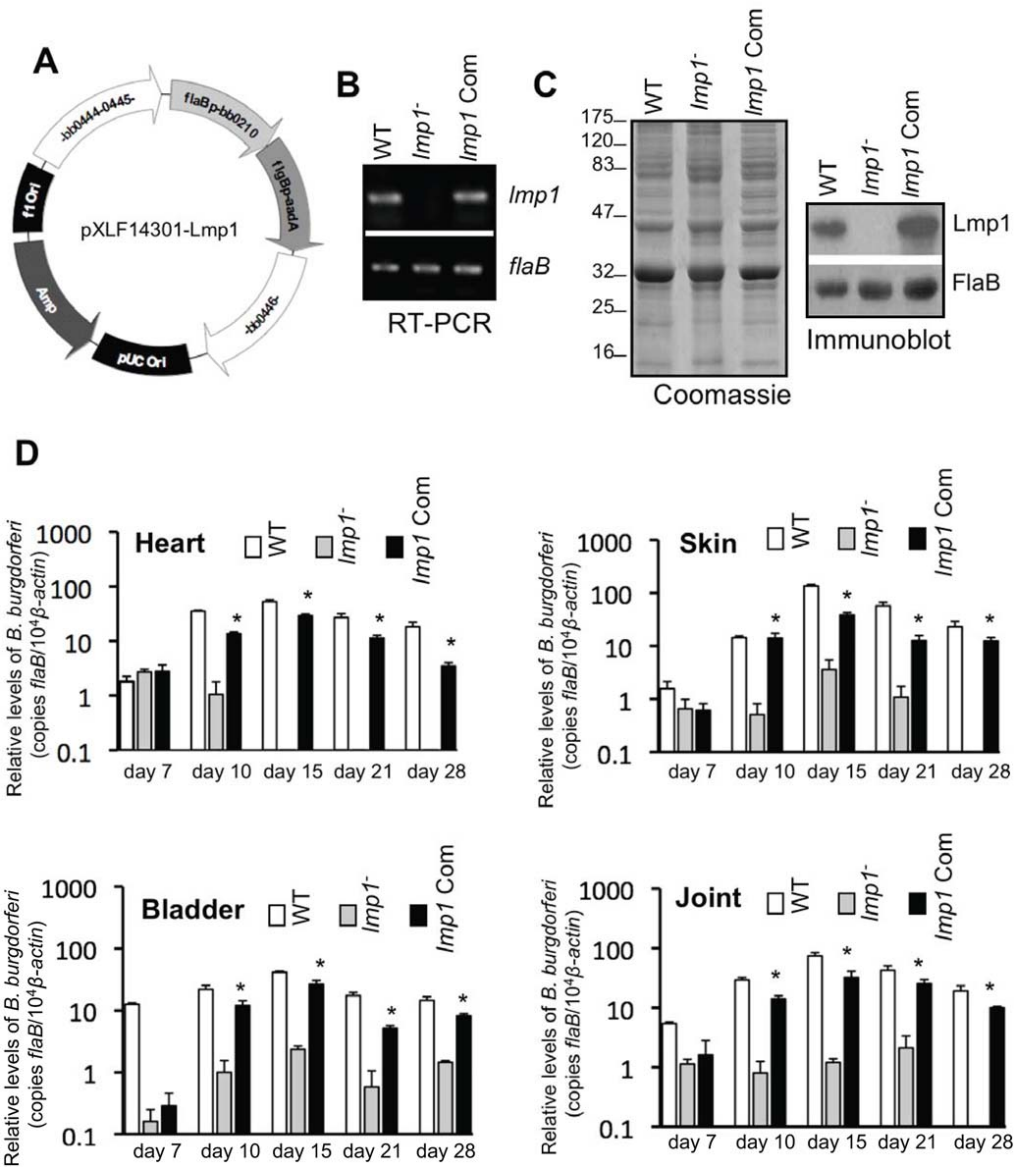
Complementation of *lmp1* mutant *B. burgdorferi* with *lmp1* gene restores the ability of spirochetes to persist in mice. (A) Construction of DNA construct (pXLF14301-Lmp1) for chromosomal integration of the *lmp1* gene. The open reading frame of *lmp1* gene was fused with *B. burgdorferi flaB* promoter and cloned into the shuttle vector pKFSS1 that houses the streptomycin resistance gene (*aadA*) under *B. burgdorferi flgB* promoter. A DNA fragment encompassing the *lmp1* gene with the *flaB* promoter and *aadA* cassette was finally excised from pKFSS1 and cloned into *BamHI* and *SmaI* sites of pXLF14301, which contains two fragments from *B. burgdorferi* chromosomal locus (*bb0444* and *bb0446*) that act as the 5′ and 3′ arms needed for homologous recombination and integration of the complemented gene in *B. burgdorferi* chromosome. (B) RT-PCR analysis of the *lmp1* transcripts. Total RNA was isolated from either the wild type (WT), *lmp1* mutant (*lmp1^−^*), or *lmp1*-complemented *B. burgdorferi* (*lmp1* Com), converted to cDNA, then subjected to PCR analysis with *flaB* and *lmp1* primers, and analyzed on a 2% agarose gel. (C) Production of the Lmp1 protein by the complemented *B. burgdorferi*. Lysates of *B. burgdorferi* were separated on a SDS-PAGE gel, which was either stained with Coomassie blue (left) or transferred to nitrocellulose membrane, and blotted with antiserum against Lmp1 or FlaB (right). (D) The *B. burgdorferi* burden in mice. Mice were infected with wild type (white bar), *lmp1* mutant (gray bar), and *lmp1* mutant complemented with *lmp1* (black bar) *B. burgdorferi*, and the spirochete burden was analyzed at days 7, 10, 15, 21, and 28 by measuring copies of the *B. burgdorferi flaB* gene, which was then normalized to mouse *β-actin* in each sample. Bars represent the mean±SEM of relative tissue levels of *B. burgdorferi* from three independent animal infection experiments. With the exception of the day 7 time point, levels of *lmp1*-complemented isolates were significantly higher than those of *lmp1* mutants in all timepoints and murine tissues (* heart, P<0.001; skin, P<0.006; bladder P<0.004, joint P<0.002).

Although these observations indicated that *lmp1* mutant *B. burgdorferi* was infectious in the murine host, the mutant was unable to establish persistent infection in mice and failed to induce disease, as assessed by the development of arthritis and carditis. To rule out the possibility that the observed phenotypic defects of the *lmp1* mutant *B. burgdorferi* to infect the murine host were the result of anomalous effects of genetic manipulation, we sought to complement the *lmp1* mutant spirochetes with a wild type copy of the *lmp1* gene *in cis*, and use this isolate in murine infection studies. As *lmp1* lacks an obvious upstream promoter, we first fused the open reading frame of *lmp1* with the *B. burgdorferi flaB* promoter. The *flaB*-*lmp1* fusion, along with the streptomycin resistance cassette, *aadA* [29] was then inserted into pXLF14301 [20] for integration into the *B. burgdorferi* chromosome (Figure 3A). *lmp1* mutants were transformed and selected using antibiotics. PCR analysis confirmed that one of the *lmp1*-complemented spirochete isolates retained all of the *B. burgdorferi* plasmids present in the parental isolate (data not shown). RT-PCR and immunoblotting showed that the *lmp1*-complemented isolate produced both *lmp1* mRNA (Figure 3B) and Lmp1 protein (Figure 3C). Lmp1 mRNA and protein production in the complemented and wild type isolates was further assessed by qRT-PCR and densitometric analysis of the immunoblot (Figure 3C), respectively, and normalized against FlaB production, which indicated that both isolates produced comparable levels of Lmp1 (data not shown).

**Figure 4:**
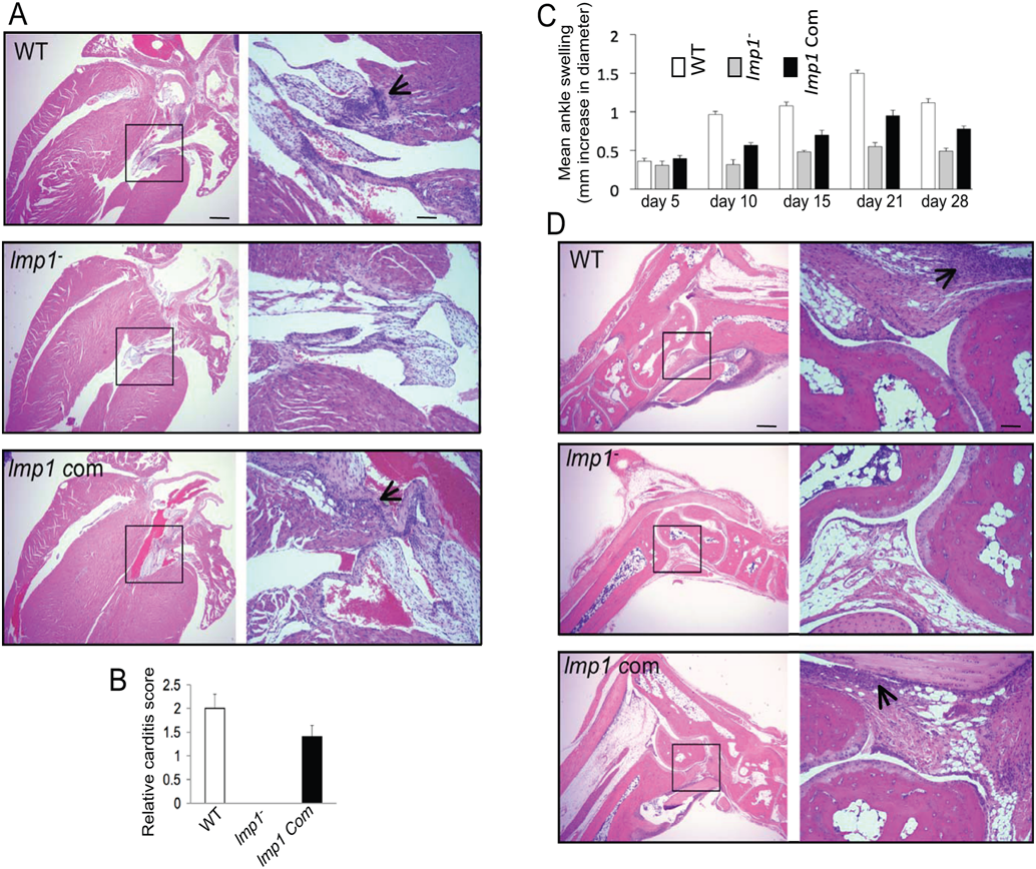
Deficiency of *B. burgdorferi lmp1* reduces the severity of carditis and arthritis in mice. (A) Representative histology of hearts isolated from mice infected with wild type *B. burgdorferi* (WT), *lmp1* mutant (*lmp1^−^*), or *lmp1* mutants complemented with *lmp1* gene (*lmp1* Com) isolates analyzed two weeks following infection. The left panel indicates lower-resolution (4×, bar = 400 µm) and higher-resolution (20×, bar = 80 µm) images of selected areas from corresponding sections (marked by box) shown in right panels. (B) Quantitative representation of the data shown in Figure 4A. At least ten random sections from each spirochete group were scored for severity of carditis in blinded fashion on a scale of 0–3 as indicated in Materials and Methods. (C) Severity of joint swelling in *B. burgdorferi*–infected mice. Arthritis was evaluated by assessment of the development of joint swelling of the mice infected with wild type *B. burgdorferi* (white bar), *lmp1* mutant (gray bar), and *lmp1* mutants complemented with *lmp1* gene (black bar) isolates, measured using a digital caliper on day 5, 10, 15, 21, and 28 following spirochetes challenge. Bars represent the mean±SEM from three independent infection experiments. Differences in the joint swelling between groups of mice infected with *lmp1* mutant and those with the *lmp1*-complemented isolates were significant (P<0.003) at all time points, except for day 5. (D) Representative demonstration of joint histology in mice infected with wild type or genetically manipulated *B. burgdorferi* isolates. Three weeks following spirochete infection, tibiotarsal joints were analyzed for histopathology. Both lower-resolution (4×, bar = 400 µm, left panel) and corresponding higher-resolution sections (20×, bar = 80 µm, marked by box, right panel) are shown. The arrows indicate the infiltration of inflammatory cells.

We then compared the ability of the wild type, *lmp1* mutant and complemented spirochetes to establish infection and induce disease in the murine host. Groups of 10 C3H/HeN mice were separately inoculated intradermally with wild type, *lmp1* mutant and *lmp1*-complemented *B. burgdorferi* (10^5^ cells/mouse). The spirochete burdens in heart, skin, bladder and joints were evaluated at day 7, 10, 15, 21 and 28 following *B. burgdorferi* infection. The results showed that, except for the initial time point (day 7), *lmp1* mutants were severely impaired in their ability to colonize all murine tissues and were undetectable in the heart after 10 days of infection (Figure 3D). Similarly, culture analysis of murine heart isolated after 15 days of *B. burgdorferi* infection showed that the *lmp1* mutant spirochetes could not be recovered (data not shown). In contrast, both wild type and *lmp1*-complemented *B. burgdorferi* readily persisted in all tested murine tissues throughout infection, with significantly higher burdens than *lmp1*-deficient spirochetes–heart (P<0.001), skin (P<0.006), bladder (P<0.004) and joints (P<0.002). Both wild type and *lmp1*-complemented *B. burgdorferi* caused severe inflammation, but *lmp1* mutants induced less severe disease, as reflected by the histopathological signs of carditis (Figure 4A and 4B), development of swelling in the tibiotarsal joints (Figure 4C), and histopathological signs of arthritis (Figure 4D). In most cases, the tissue burdens of *lmp1*-complemented isolates (Figure 3D) and their ability to induce inflammation (Figure 4B and 4C) were comparable, yet significantly lower (P<0.05), than those of the wild type isolates, possibly due to the constitutive expression of *lmp1* from the heterologous *flaB* promoter which might be detrimental to spirochetes.

### *lmp1* contributes to *B. burgdorferi* survival against host-acquired immune defenses

**Figure 5:**
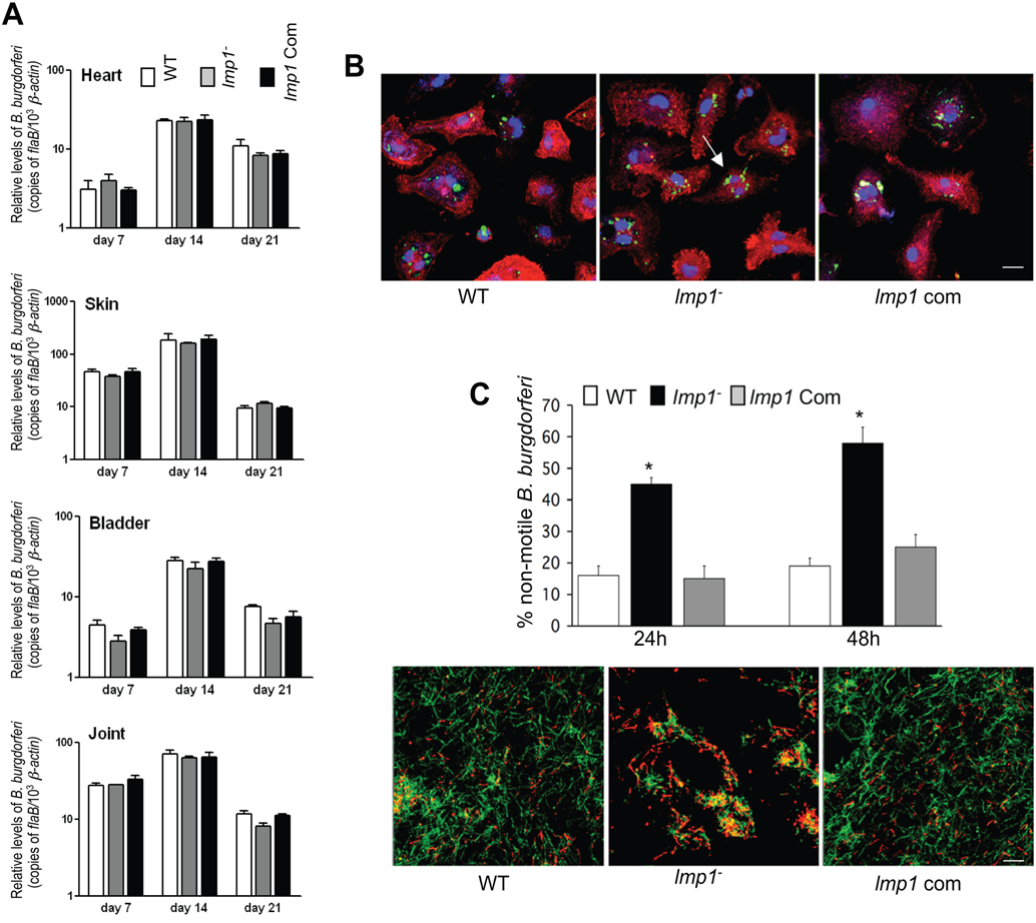
Lmp1 contributes to *B. burgdorferi* defense against host-acquired immune responses. (A) *lmp1* deletion does not influence *B. burgdorferi* infectivity in severe combined immunodeficient (SCID) mice. Spirochete burdens in murine tissues (heart, joint, skin, and bladder) were analyzed at days 7, 14, and 21 post-infection with wild type (white bar), *lmp1* mutant (gray bar), and *lmp1* mutant complemented with *lmp1* (black bar) *B. burgdorferi* and presented as copies of the *B. burgdorferi flaB* gene normalized against mouse *β-actin*. Bars represent the mean±SEM from two independent infection experiments. Levels of *B. burgdorferi* isolates are similar in all murine tissues and timepoints (P>0.05). (B) Uptake of *B. burgdorferi* by primary murine macrophages. Bone marrow-derived macrophages were isolated from C3H mice and incubated with wild type *B. burgdorferi* (WT), *lmp1* mutant (*lmp1^−^*), and *lmp1* complemented (*lmp1* Com) isolates at an MOI of 10. Cells were fixed after 2 hr of spirochete addition and processed for confocal immunofluorescence microscopy. Engulfed spirochetes (arrow), cellular actin, and nuclei were detected using FITC-labeled anti-*B. burgdorferi* goat IgG, phalloidin-Texas Red, and DAPI, respectively. A representative image of three independent experiments with similar results is presented (bar = 20 µm). (C) The *lmp1* mutant is highly susceptible to anti-*B. burgdorferi* antibody-mediated killing *in vitro*. The wild type *B. burgdorferi* (WT), *lmp1* mutant (*lmp1^−^*), and *lmp1* complemented (*lmp1* Com) isolates were incubated in the presence of *B. burgdorferi*–infected mouse serum. Serum used in the assay was pooled from *B. burgdorferi*–infected mice following 15 days of syringe-based infection (10^5^ cells/mouse). Upper panel represents spirochete viability after 24 and 48 hrs of additional *B. burgdorferi*–infected serum, as assessed by the increase in the number of non-motile spirochetes using dark-field microscopy. Results are mean±SEM from three representative experiments with similar results. Differences between *lmp1* mutant and wild type *B. burgdorferi* or *lmp1*-complemented spirochetes were highly significant (*P<0.002). The lower panel shows a representative fluorescence labeling of the live and dead spirochetes after 48 hr of antiserum treatment. Live bacteria were stained green (Syto 9 stain) whereas the dead bacteria stained red (propidium iodide).

The analysis of spirochete growth in the culture media indicated that the wild type, *lmp1* mutants and *lmp1*-complemented isolates follow a similar growth pattern without significant variation in motility or obvious morphological defects (data not shown). However, *lmp1* mutants were impaired to persist in the immunocompetent murine hosts following the first week of infection. We next assessed whether Lmp1 function *in vivo* is related to the metabolic or immune environment of the host. To accomplish this, we compared the infectivity of wild type and *lmp1* mutant *B. burgdorferi* in the established immunodeficient murine model of Lyme borreliosis using severe combined immunodeficient (SCID) mice [30]. Groups of SCID mice (3 animals/group) were inoculated intradermally with 10^5^ wild type, *lmp1* mutant, or *lmp1*-complemented *B. burgdorferi*. The spirochete burdens in the heart, skin, bladder, and joints were evaluated at day 7, 14 and 21 following *B. burgdorferi* infection using quantitative PCR. In parallel, the viability of the spirochete was determined by culture of murine blood and spleen isolated at day 7, 14 and 21. The results showed that the wild type, *lmp1* mutant and *lmp1*-complemented *B. burgdorferi* could be cultured from murine tissues at all time points (data not shown) and that there was no significant difference in *B. burgdorferi* burdens in all tested murine tissues throughout the infection (Figure 5A). Consistent with similar burdens of wild type and genetically-manipulated pathogens in SCID mice, one of the innate immune mechanisms, the ability of macrophages to phagocytose invading pathogens, which is important in controlling *B. burgdorferi* infection, did not differ between wild type and *lmp1* mutants (Figure 5B). As neutralizing antibodies that develop in *B. burgdorferi* infected mammals are primarily responsible for controlling spirochete burden in the host, we explored whether reduced virulence of *lmp1*mutant spirochetes correlates with the susceptibility of *B. burgdorferi* to the borreliacidal activity of immune sera. To accomplish this, wild type, *lmp1* mutant and *lmp1*-complemented *B. burgdorferi* were exposed to antisera collected from *B. burgdorferi*–infected C3H mice. *lmp1* mutants were significantly more susceptible to the bactericidal activities of the anti-*B. burgdorferi* sera than wild type *B. burgdorferi*, and were protected by genetic complementation with *lmp1* (Figure 5C). The susceptibility of *lmp1* mutants to the bactericidal activities of the immune sera did not differ significantly when using active or heat-inactivated serum (data not shown), indicating that *lmp1* deletion enhances the borreliacidal effects of antibodies in a complement independent manner. Like parental isolates, the *lmp1* mutants were not susceptible to bactericidal activities by the non-immune serum collected from naïve mice (data not shown), suggesting that Lmp1 is not required for serum resistance by spirochetes. Together, these data suggest that Lmp1 contributes to *B. burgdorferi* defense against host-acquired immune responses by enhancing resistance to the bactericidal antibodies that develop during infection.

## Discussion

In nature, *B. burgdorferi* is maintained through a complex enzootic cycle [1]. Once transmitted to mammals, *B. burgdorferi* can establish persistent infection in a variety of tissue locations. Limited studies suggest that spirochete genes expressed in higher levels in infected host tissues could be important for *B. burgdorferi* survival [8]. Since membrane proteins could have a direct contribution to the adaptation of pathogens to host environments, we assessed the expression of 91 *B. burgdorferi* genes encoding potential membrane proteins covering spirochete infectivity in multiple murine tissues. Our data show that few of the genes analyzed are differentially or highly expressed in the selected tissues. Targeted deletion of one of the spirochete genes that is highly expressed in cardiac tissue, *lmp1*, while resulting in the initial clearance of pathogen burden in infected hearts, also affected overall virulence of *B. burgdorferi* in murine infectivity and reduced the outcome of Lyme disease. Our results show that *lmp1* mutants persist in SCID mice at similar levels to parental isolates and are susceptible to borreliacidal antibody-mediated killing *in vitro*, suggesting that Lmp1 contributes to *B. burgdorferi* defense against host-acquired immune responses. Identification of hitherto unrecognized virulence genes of *B. burgdorferi*, such as *lmp1*, that support pathogen infectivity in mammals could shed light on the pathogenesis and prevention of Lyme disease.

Genes that are selectively expressed *in vivo* could be important for *B. burgdorferi* persistence in nature, possibly allowing spirochete adaptation to highly heterogeneous metabolic and immune environments. While assessment of pathogen gene expression *in vivo* is an important prerequisite to understanding microbial pathogenesis, microarray analysis is of limited use for the assessment of the *B. burgdorferi* transcriptome *in vivo*, primarily due to the low level of pathogen RNA in infected tissues [31]. Microarray studies were attempted after isolation and amplification of microbial RNA [7],[31], but this requires longer periods of RNA manipulation and risks degradation or clonal alteration during amplification and processing of transcripts. In contrast, optimal quantities of *B. burgdorferi* RNA can be isolated from spirochetes grown *in vitro* or in a host-implanted dialysis membrane, and microarray-based studies have been used to assess *B. burgdorferi* gene expression in these ‘host-like’ conditions that have yielded important information on the role of *B. burgdorferi* genes in pathogen infectivity [9],[12],[13],[15],[32]. However, the host environment is too complex and dynamic to be duplicated artificially. Therefore, we employed a sensitive qRT-PCR approach for the direct assessment of pathogen gene expression *in vivo*. This method is reproducible, as two independent sets of animal infection studies identified the same set of 47 *B. burgdorferi* genes as expressed *in vivo*, and a majority of them displayed higher expression levels in mice, than *in vitro*. The variable expression of these genes in multiple tissue locations possibly reflects the adaptive responses of the pathogen to local host environments, enabling immune evasion, adhesion, or nutrient uptake, among other possibilities. Furthermore, regardless of their functional role in pathogen persistence, antigens that are highly produced in certain host sites, such as the joints and heart, could participate in the genesis of inflammatory disease. Our qRT-PCR analysis also identified a set of 44 *B. burgdorferi* genes that may not be important for mammalian infectivity, as none of these displayed detectable transcription within the first 4 weeks of infection. On the other hand, a set of 26 genes encoding potential lipoproteins displayed detectable expression *in vivo* and, with the exception of *bbo40*, expression of many genes (*bb0806*, *bbb09*, *bbd10*, *bbj09*, *bbl39*, *bbm27*, *bbn38*, *bbn39*, *bbq47*, *bbs41*) agreed with a previous study that evaluated the expression of *B. burgdorferi* lipoproteins in murine dermis [5]. Although our expression analysis identified selected *B. burgdorferi* transcripts highly expressed *in vivo*, rather than proteins, these mRNA are likely the signatures of translated antigens. This speculation is supported by the recent study that identified 103 spirochete immunogens by screening *in vitro* translated genome-wide proteomic arrays with *B. burgdorferi*-specific immune sera [33]. Our target list of potential membrane proteins (Table S1) overlaps 10 of these identified immunogens (BB0543, BBB09, BBH06, BBL39, BBM27, BBN38, BBN39, BBO39, BBO40 and BBS41). Each of these immunogens was represented in our identified set of *B. burgdorferi* genes expressed in murine tissues (Figure S1).

Our study established *B. burgdorferi lmp1* as a spirochete gene that is highly expressed in the early stages of mammalian infection, most notably in the murine heart. Lmp1 was first described in an earlier study [34] which highlighted the protein as a potential *B. burgdorferi* adhesin and indicated that Lmp1-specific antibodies develop in *B. burgdorferi*–infected mammals. *lmp1* does not belong to the paralogous gene families in *B. burgdorferi* [24] and is thought to encode a relatively large 128-kDa outer membrane protein with putative type I signal peptide. It is not clear if the leader peptide is indeed cleaved or if the hydrophobic N-terminus serves as a membrane anchor; however, the antigen is exposed on the spirochete surface. Lmp1 retains 86–88% amino acid identity across orthologs in the related infectious spirochetes *B. afzelii* and *B. garinii*, with the highest sequence conservation in the amino and carboxyl termini. Analysis of the *lmp1* locus indicates that the gene overlaps with the immediate upstream gene, *bb0209*, and shares a short intergenic region with the downstream gene, *bb0211*, suggesting these three genes are likely part of an operon; however, the *lmp1* mutant is able to express both *bb0209* and *bb0211*. Notably, *bb0211* encodes for the DNA mismatch repair protein, MutL, and therefore likely bears a house-keeping function in *B. burgdorferi* biology. Although the functions of *bb0209* and *lmp1* are unknown, the amino terminus of BB0209 and the carboxyl terminus of Lmp1 both contain multiple tetratricopeptide repeat domains [35], which are shown to mediate protein-protein interaction and the assembly of the pilus and multiprotein complexes [36]. In addition, the central region of Lmp1 harbors a unique cluster of seven repeat motifs, each consisting of 54 amino acids, which is highly conserved amongst Lmp1 orthologs and potentially participates in adherence to host components [34]. Our studies further establish that Lmp1 is a surface-exposed virulence factor of *B. burgdorferi*, as *lmp1* deletion severely interferes with spirochete infectivity and pathogenesis. After its inoculation in the host, *B. burgdorferi* remains locally in the dermis for a few days and then disseminates to distant organs, likely via the bloodstream, during the first week of infection [37],[38]. Our data indicate that Lmp1 function may not be important during early infection, including spirochete dissemination, since no apparent differences in the burdens of wild type spirochetes and *lmp1* mutants were observed in murine blood samples collected at day 3, 5 (data not shown) and 7 (Figure 2G). Instead, *lmp1* mutants displayed a severe reduction in numbers in the disseminated organs by day 10, implying that Lmp1 is critical for the persistence of the spirochete in murine tissues. In agreement with the higher level of expression of *lmp1* in the cardiac tissue, as compared to other organs, a greater effect of *lmp1* deletion was also observed in the heart where the mutants were selectively eliminated after 10 days of infection, suggesting that Lmp1 plays a dominant role in spirochete infection of the heart. *B. burgdorferi* burdens in mice began to decline after first two weeks of infection [37], which coincides with the development of the acquired immune response, such as neutralizing antibodies that controls spirochete infection [26],[28]. This is consistent with the observation that *lmp1* mutants persist in similar levels to the parental isolate in the heart at day 7, despite dramatic wild type expression of *lmp1*, but begin to decline during the second week. The ability of *lmp1* mutants to survive in SCID mice further indicates that the function of the Lmp1 is not related to metabolic requirements of the spirochete survival *in vivo*, as shown for *B. burgdorferi* PncA [39] or AdeC [40], but rather is associated with *B. burgdorferi* survival in host immune environments. *B. burgdorferi* isolates missing the lp28-1 plasmid [41], which houses VlsE [42],[43], display similar impaired host persistence in immunocompetent mice, as VlsE likely confers protection against host-generated borreliacidal antibodies. Therefore, while *lmp1* is variably expressed in diverse tissue environments and carries a predominant role in spirochete persistence in the heart, a basal level of *lmp1* expression is noted throughout the murine infection which, based on our *in vitro* data (Figure 5C), might contribute to the protection of *B. burgdorferi* against host-acquired immune responses, as was recently proposed for *B. burgdorferi* OspA in feeding ticks [44].

In summary, we have identified a select set of *B. burgdorferi* genes encoding potential membrane proteins that are expressed during murine infection. Many of these *in vivo*-expressed genes are differentially expressed in various host tissues, including joints and heart, and can participate in pathogen persistence and the genesis of disease. Here, we present direct evidence that one microbial gene expressed at higher level in the cardiac tissue, *lmp1*, encodes an essential virulence factor that plays an important role in immune evasion and dramatically influences spirochete persistence in murine tissues and the genesis of inflammation. Whereas previously identified *B. burgdorferi* virulence antigens are mostly plasmid-borne, and thus have greater instability and sequence divergence, Lmp1 is chromosomally encoded and is relatively conserved among orthologs in related infectious spirochetes. Further identification of *B. burgdorferi* virulence determinants that actively support spirochete persistence *in vivo* could contribute to the development of effective therapeutic strategies against Lyme borreliosis.

## Materials and Methods

### Bacteria, mice, and ticks

*Borrelia burgdorferi* infectious isolate A3 [45], a clonal derivative of the *B. burgdorferi* whole genome sequenced strain B31 M1 [24],[25], was used in this study. Four- to six-week old female C3H/HeN and pathogen-free NCr-SCID mice were purchased from the National Institutes of Health. Mice were inoculated with a single subcutaneous injection of 10^5^ spirochetes per mouse. All animal procedures were performed in compliance with the guidelines and with the approval of the Institutional Animal Care and Use Committee. *Ixodes scapularis* ticks used in this study belong to a colony that has been reared and maintained in the laboratory.

### Quantitative RT-PCR analysis

The identity and oligonucleotide primer sequences for the quantitative RT-PCR analysis of *B. burgdorferi* genes are indicated in Table S1. The *B. burgdorferi* target genes [24],[25]were selected based on their predicted localization on the spirochete membrane according to the database annotation (www.tigr.org) and PSORT *in silico* analysis [46]. Groups of mice (5 animals/group) were infected with *B. burgdorferi* (10^5^ spirochetes/mouse), and samples of skin, heart, tibiotarsal joint and bladder were collected and frozen in liquid nitrogen at one-week intervals between 1 and 4 weeks of infection. Total RNA was extracted from tissue samples using the TRIzol reagent (Invitrogen). To reduce traces of contaminating DNA, samples were further digested with RNase-free DNaseI (Qiagen), purified using the RNeasy kit (Qiagen) and reverse transcribed to cDNA using the AffinityScript cDNA synthesis kit (Stratagene). The relative levels of *B. burgdorferi* cDNA in each sample were assessed by quantitative PCR (qPCR), and DNA contamination in each sample was measured using an equal volume of purified RNA as a template. Samples from each time point were pooled by tissue type, and final pools of skin, heart, joints and bladder were used in the qPCR analysis. The primers used for qPCR reaction were designed using OligoPerfect Primer design software (Invitrogen) based on the *B. burgdorferi* B31 M1 genomic sequence [24],[25]. All PCR primer pairs had a similar annealing temperature (60°C) and spanned 100–300 base pairs of each of the target *B. burgdorferi* genes. Each primer pair was tested for efficiency and non-specific amplification by melt-curve analysis using *B. burgdorferi* genomic DNA as a template. In one case of paralogous genes, the same set of primers was assigned for the detection of both genes as indicated in Table S1. To generate reliable *in vivo* gene expression data and to further ensure specific amplification of *B. burgdorferi* cDNA in murine tissue samples, the qPCR amplification in each well was followed by melt-curve analysis, and wells showing non-specific amplification were discarded from data analysis. The amplification cycle consisted of initial denaturation at 95°C for 5 min followed by 45 cycles each at 95°C for 10 sec, 60°C for 20 sec and 72°C for 30 sec and final melt curve analysis: 55°C for 30 sec, increase 0.5°C per cycle to 95°C. The amplification was performed in an iQ5 real-time thermal cycler (Bio-Rad) using SYBR Green Supermix (Bio-Rad) as detailed. For expression screening of *B. burgdorferi* genes, we simultaneously assayed 8 candidate genes in each 96-well PCR plate using duplicate wells of template cDNA (skin, heart, joint and bladder samples) with parallel positive (*B. burgdorferi* genomic DNA) and negative (no template) controls. Transcript levels of individual genes were assessed in spirochetes grown *in vitro* in BSK medium (10^7^ cells/ml) and in each of the murine samples, calculated using the 2^−ΔΔCt^ method [47], normalized against *flaB* transcripts and presented as fold increase in gene expression. Two independent mouse experiments used the same parameters of gene expression analysis to ensure the reproducibility of the assay. For detailed temporal and spatial analysis of *lmp1* expression by qRT-PCR analysis, amounts of target transcripts were calculated from standard curves prepared from known quantities of *flaB* and *lmp1* DNA as described [21],[48]. Mice (5 animals/group) were infected via single intradermal injection with *B. burgdorferi* (10^5^ spirochetes/mouse) or via tick feeding using *B. burgdorferi*–infected nymphs. Infected murine samples, the heart, skin, bladder and tibiotarsal joints, were removed at different timepoints and frozen in liquid nitrogen. *B. burgdorferi*–infected ticks were isolated by allowing ticks to feed on 15-day infected mice as described [7]. For quantitative measurement of *B. burgdorferi* burden in infected tissues, *flaB* transcripts were measured in infected samples and normalized to mouse or tick *β-actin* levels.

### Generation and phenotypic analysis of genetically manipulated *B. burgdorferi* isolates

The oligonucleotide primers used for mutagenesis and genetic complementation of *B. burgdorferi* are indicated in Table S2. The *lmp1*-deficient *B. burgdorferi* was created by exchanging a 2068 base pair DNA fragment encompassing the 5′ terminus of the *lmp1* gene with a kanamycin-resistance cassette via homologous recombination as described [8]. Briefly, DNA fragments flanking up- and downstream of the *lmp1* gene were PCR-amplified using primers P1–P4 and inserted into two multiple-cloning sites flanking the kanAn cassette in plasmid pXLF10601 [49]. This plasmid was sequenced to confirm the identity of the insert and electroporated into wild type *B. burgdorferi* B31 isolate A3. Transformants were selected for growth in the presence of kanamycin (350 µg/ml). Ten clones were isolated and PCR analysis was used to confirm the intended recombination event using primers P1–P9. The presence of all endogenous plasmids contained in the parental A3 isolate was also assessed in the mutant clones as described [8]. One of the *lmp1* mutant clones that retained the same complete set of plasmids as the wild type isolate was used in additional experiments.

Genetic complementation of the *lmp1* mutant was achieved by re-insertion of a wild type copy of the *lmp1* gene in the *B. burgdorferi* chromosome [20]. The upstream of the *lmp1* open reading frame (ORF) overlaps with the preceding gene by a few nucleotides, lacking an intergenic region with a discernible promoter. We, therefore, fused the *lmp1* ORF with the *B. burgdorferi flaB* promoter [50]. Two *B. burgdorferi* DNA fragments encompassing the full-length *lmp1* gene and the *flaB* promoter were PCR-amplified, fused and cloned into the *BamHI* and *SalI* sites of pKFSS1 housing a streptomycin-resistance cassette *(aadA)* [29]. A DNA element containing the *flaB* promoter-*lmp1* gene fusion and the *aadA* cassette was cut with *BamHI* and *SmaI* from the recombinant plasmid pKFSS1-lmp1 and inserted into the corresponding restriction sites of the plasmid pXLF14301 [20] that contains the required 5′ and 3′ arms for homologous recombination in the *B. burgdorferi* chromosomal locus *bb0444*–*0446*. The plasmid construct was sequenced to confirm its identity and 25 µg of the plasmid DNA was electroporated into the *lmp1* mutant. Four clones were isolated by their ability to grow in the presence of both kanamycin and streptomycin. PCR analysis was used to confirm the intended recombination event, and one of the *lmp1*-complemented clones that contained the same plasmid profiles as the wild type was chosen for further study. For *in vitro* growth analysis, an equal number of wild type and genetically-manipulated spirochetes were diluted to a density of 10^5^ cells/ml and grown at 33°C in BSK-H medium until they reached the stationary phase (10^8^ cells/ml). Aliquots of spirochetes were assessed every 12 hours, under a dark-field microscope, for motility, cell clumping and numbers of spirochetes counted using a Petroff-Hausser cell counter.

For phenotypic analysis of *lmp1* mutants and *lmp1*-complemented isolates *in vivo*, *B. burgdorferi* were injected into groups of mice (10 animals/group) via needle-inoculation (10^5^ spirochetes/mouse) intradermally on the back. Mice were sacrificed at 7, 10, 15, 21 and 28 days following inoculation. Skin, heart, joints, bladder and blood samples were collected and *B. burgdorferi* burdens were measured by quantitative PCR analysis as previously described [7],[8]. Development of joint swelling in the infected mice was also evaluated at similar time points as for spirochete burdens. For histological evaluation, heart and joint samples were collected at days 15, 21 and 28 following inoculation. For tick acquisition studies, groups of mice (5 animals/group, 20 ticks/mouse) were fed on by naïve ticks following two weeks of *B. burgdorferi* infection. The ticks were allowed to feed to repletion and were immediately analyzed for quantitative RT-PCR measurement of *B. burgdorferi* burden as detailed earlier [7].

### Antibodies, ELISA, and immunoblotting

Generation of murine polyclonal antibodies against recombinant Lmp1, ELISA and immunoblotting were performed as described [7],[8]. Recombinant Lmp1 protein was produced in *E. coli* using the bacterial expression vector pGEX-6P1 (Amersham-Pharmacia Biotech) with specific primers as indicated in Table S2. Expression, purification and enzymatic cleavage of the glutathione transferase (GST) fusion proteins were carried out as detailed [7],[8]. Sixteen serum samples from humans with a clinical history of Lyme disease, collected from the CDC Lyme patient serum panel were used in the ELISA. Five serum samples from normal individuals residing in non-endemic areas for Lyme disease were also collected from CDC and used as negative controls. For immunoblotting, recombinant Lmp1 (0.05 µg/lane) was resolved on a SDS-PAGE gel and probed with 1∶ 1000 dilution of murine or human serum. Murine antiserum was collected from a group of 5 mice, 15 days after infection with *B. burgdorferi* via syringe inoculation (10^5^ cells/mouse). Murine antiserum generated against recombinant Lmp1 was used in a Proteinase K accessibility assay to determine surface exposure of the Lmp1 as described [48].

### Phagocytosis and *in vitro* killing assay

Bone marrow derived macrophages were isolated from naïve C3H mice as described [51] and cultured for 5 days at 37°C (2×10^5^ cells/well) in L929-conditioned DMEM media. The cells were then washed and resuspended in serum-free DMEM and incubated with *B. burgdorferi* at a multiplicity of infection (MOI) of 10 at 37°C for 2 hours. Cells were washed with cold PBS to remove unbound *B. burgdorferi* and fixed in 3.7% paraformaldehyde, and processed for confocal immunofluorescence as described [52]. Spirochetes, cellular actin and nuclei were detected using FITC-labeled anti-*B. burgdorferi* goat IgG (KPL), phalloidin-Texas Red (Invitrogen), and DAPI (Invitrogen), respectively.

Susceptibility of wild type or genetically manipulated spirochetes to borreliacidal activities in infected mouse sera was performed as described [53]. Briefly, spirochetes were grown in BSK-H medium to a density of 10^7^ spirochetes/ml and exposed to active or heat-inactivated 50% sera isolated from mice infected for 15 days with *B. burgdorferi*. Samples were incubated at 33°C for 24 to 48 hours and spirochete viability was assessed using dark-field microscopy. Susceptibility of spirochetes to borreliacidal activities in infected mouse sera were also tested in parallel using a combination of two vital stains that specifically label live and dead spirochetes, as described [8]. Briefly, spirochetes were incubated for 48 hours and labeled with the live/dead *Bac*Light Viability kit (Invitrogen) according to the manufacturer's instructions.

### Evaluation of arthritis and carditis

*B. burgdorferi*–infected mice were examined for swelling of the tibiotarsal joints as detailed earlier [8]. Ankle joints from each of the rear legs of each mouse were measured using a precision metric caliper in a blinded fashion. The thickest diameters of the tibiotarsal joints were measured in each mouse prior to *B. burgdorferi* infection, and the development of ankle swellings was monitored and tabulated on a weekly basis until the sacrifice of the mice. For histological evaluations of arthritis and carditis, at least 5 ankle joints and 5 hearts were collected from each group of mice (5 animal/group) infected with the different isolates. For histology, joints and hearts (cut in half through bisections across the atria and ventricles) were fixed in 10% formalin and processed for Hematoxylin and Eosin staining. Twenty randomly chosen sections from each mouse group were assessed for histopathological comparisons. Signs of arthritis were evaluated as described [8], based on a combined assessment of histological parameters of *B. burgdorferi*-induced inflammation [54]–[56], such as exudation of fibrin and inflammatory cells into the joints, alteration in the thickness of tendons or ligament sheaths, and hypertrophy and hyperplexia of the synovium. Signs of carditis [54],[57] were evaluated based on the cardiac inflammatory infiltrate, including the transmural infiltration of neutrophils in the blood vessels and infiltration of surrounding connective tissue with macrophages. Carditis was scored on a scale of 0 (no inflammation), 1 (mild inflammation with less than two small foci of infiltration), 2 (moderate inflammation with 2 or more foci of infiltration), or 3 (severe inflammation with focal and diffuse infiltration covering a large area). Both joint and heart tissue sections were blindly examined by two independent researchers.

### Statistical analysis

Results are expressed as the mean±standard deviation (SD) or standard error mean (SEM). The significance of the difference between the mean values of the groups was evaluated by two-tailed Student *t* test.

## Supporting Information

Figure S1 Relative expression of selected *B. burgdorferi* genes during infection of the murine hosts. Total RNA was isolated from *B. burgdorferi* grown *in vitro*, and multiple tissues of mice, between 1 and 4 weeks of *B. burgdorferi* infection, were pooled by tissue type and converted to cDNA for measuring gene-specific transcripts using quantitative PCR. Fold increase in the expression of individual genes in each of the murine samples was calculated based on threshold cycle (Ct) values using the 2^−ΔΔCt^ method [47], normalized against *flaB* Ct values. Bars represent the mean±SD from four quantitative PCR analyses of two independent infection experiments.
(0.8 MB EPS)

Figure S2 *lmp1* is highly expressed in the murine heart during early phases of tick-borne *B. burgdorferi* infection. Total RNA was isolated from multiple murine tissues following 7 days of challenge with *B. burgdorferi*–infected nymphal ticks (5 ticks/mouse, 3 animals/group), converted to cDNA, and used for measuring *lmp1* transcripts in quantitative PCR assay. The relative expression levels of *lmp1* are presented as copies of *lmp1* transcript per 1,000 copies of *flaB* transcripts. Bars represent the mean±SEM from three independent experiments. The transcript levels of *lmp1* in the heart were significantly higher than corresponding expression levels in the skin, joint, or bladder (*P<0.02).
(0.4 MB EPS)

Figure S3 Recognition of *B. burgdorferi* Lmp1 by immune sera. (A) Development of Lmp1-specific antibody response in *B. burgdorferi*–infected mice and humans. Fifty nanograms of recombinant Lmp1 was probed with normal and *B. burgdorferi*–infected sera. Arrow indicates the development of Lmp1-specific antibody response in both *B. burgdorferi*–infected mice and human sera. Infected serum used for immunoblotting was pooled from infected mice following 15 days of syringe-based infection or from a Lyme disease patient as detailed in the text. (B) Reactivity of antibodies in human sera to recombinant Lmp1 as assessed by ELISA. Sera from randomly chosen normal healthy controls (n = 5) and Lyme disease patients (n = 16) were tested for detection of antibodies specific for recombinant Lmp1.
(0.3 MB EPS)

Figure S4 Detection of native *B. burgdorferi* Lmp1. Murine antibodies generated against Lmp1 specifically recognize native protein in *B. burgdorferi*. One microgram of *B. burgdorferi* lysates was probed with normal mouse serum (NMS) or murine anti-serum against Lmp1 (anti-Lmp1). Murine polyclonal antibodies used in the immunoblotting were generated by immunization of mice against recombinant Lmp1 in mice as described in the text. Arrow indicates detection of native *B. burgdorferi* Lmp1.
(0.3 MB EPS)

Table S1 Oligonucleotide primers used in the study
(0.2 MB DOC)

Table S2 Oligonucleotide primers used in the study
(0.07 MB DOC)
