# Supplementary material for: A Chromosomally Encoded Virulence Factor Protects the Lyme Disease Pathogen against Host-Adaptive Immunity
Source: PLoS Pathog. 2009 Mar 6;5(3):e1000326. doi: 10.1371/journal.ppat.1000326 (PMC2644780; doi:10.1371/journal.ppat.1000326)
Supplement: Table S1 — Oligonucleotide primers used in the study (0.2 MB DOC) [file ppat.1000326.s005.doc]

**Table S1**: Oligonucleotide primers used in the current study

| **ORF** | **Putative Function or Gene Symbol** | **Paralog**  **Family** | **Sequence (5′ → 3′: Forward, Reverse)** |
| --- | --- | --- | --- |
| BB0006 | CHP | none | ttgtttgggcagttctttcc, tcttttcccctgcatttttg |
| BB0017 | CHP | none | GGATTTGGCAATCAAAAAGC, CCAAGCACCTTTTCCGTCTA |
| BB0034 | HP | 48 | GGGATGAGTGCTGGAGAAAA, TCCTTGAGCAAAGGAGCCTA |
| BB0037 | plsC | none | gggaaatcatattgcggcta, tcggggaaaattccaataga |
| BB0051 | CHP | 47 | TTTGGAAAATCTGGGCTTTTT, ACCACGGATTTTGTTGCTTC |
| BB0073 | HP | none | TCAAACGATTTCATGCTTTCA, TTGATTTTGGGGTTTTTATCA |
| BB0090 | V-type ATPase | none | cagcaataggttctgcgttg, agcaatagcaaacccaccac |
| BB0124 | HP | none | TTTTGTTGCGTTTTTAAACATATTG, CGACCCCAAAATAGGAAACA |
| BB0157 | HP | none | TAACCAGCGCCCTTGAAATA, TCCAATTATTTGCTGATTGCTTT |
| BB0164 | Na+/Ca+ Exchange | none | AGACATTGCATTTGGCAACA, AAATGCCCCTACCAAAAACC |
| BB0165 | HP | none | CACGCCCTTTGGAATAAAAA, TCAACGGGTTGCATCTCATA |
| BB0170 | HP | none | AAACCGATTCCTGGAGAGGT, TGGAAAATTCCCTTGACCTG |
| BB0173 | HP | 121 | TCGCTTTACGGCAATTTCAACTC, ACAATGTCAGCACCAGCACTAAGA |
| BB0193 | LP | none | CGAAGACCTTGAATACTTGGAAA, AAATCAGGCTCTTTAAGGGTGA |
| BB0202 | hemolysin | 42 | AAAAGGGGAAATCGGGAATA, TGATATTGCAAGTGCGCTGT |
| BB0210 | lmp1 | none | AATGACGCTCAGGCCTCTAA, TTGTTGGCGTGATTTTGTGT |
| BB0213 | LP | none | TTTGCTTGTTCTTGCGACAT, TCCTTTCCAAGGCTAGAATCA |
| BB0234 | CHP | none | GGCAACGGTTCAAGTGAATTA, TGAAAAATCCCCCAACAAAA |
| BB0303 | mraY | none | AGGGCTGATTTTGCAGCTTA, AAAAACACCCGCAAGAATTG |
| BB0309 | HP | none | GGCGAATTTACATCAAATGCT, GAGCCTGAGGCATTTCAATC |
| BB0311 | CHP | none | TACGCCTATTTCTCCACATTCT, ATTCAAAAACAACGTCAACTCC |
| BB0316 | CHP | 129 | TTGGGCGAGAAAATAAGCAGT, GCAAATAGCAAGCCAACCA |
| BB0360 | HP | none | CAAAGCCTTTACAGAGCAGGA, GGCCATGAAAAATTCAGGTG |
| BB0361 | ylxH-2 | 32 | CTGGCAAACGAAGGAAAAAG, AAAAGCTGTTCCTGCTCCAA |
| BB0373 | HP | none | GGGTGAAATATTGTGAGGCTCT, TGCTTTCAAGCAAATCAAAAGA |
| BB0378 | HP | none | GGATGATCAAGCATATTTGAAGC, ACCCAATCACGCGAATCTAC |
| BB0398 | LP | none | TCAAAGCTGGGATCAAAAGG, ACACGCCTATCCAAGTCCA |
| BB0406 | HP | 35 | CGGATTGGTCAAAAACATCC, GTTGGCCCTACTGCAGGATA |
| BB0412 | HP | none | GCCAAAACCACGGAATTCTT, GATTGGGAGAAAGGCATAAGG |
| BB0418 | HP | none | AAAAACAAAGGCTGGGGAAG, CCAGTCCGGCTGTGTTACTT |
| BB0451 | chromate transport | 29 | TTAAATTTCGGAGGCGGTAA, GCCCAGGGGTTATTCTTGAT |
| BB0452 | HP | 29 | CCGCTCAACAACAAAATGAA, CCTCCGCCAATTGTAAGTGT |
| BB0460 | LP | none | GATGCAAAAATTCCTTTTATGG, TTTTGAGTTTTCGGGGTTTG |
| BB0473 | CHP | 56 | TTTGTCAGCGCTTTCACTTG, CTCCCCTTTTACACCCAACA |
| BB0475 | LP | none | CTTGCATGAAAACAAGCACAA, TTGCCAATGAATTTGCATTTA |
| BB0509 | HP | none | TTGCTGATGGTGCAATGAAT, TCGAATCCGCTACCCATTTA |
| BB0527 | CHP | none | GGAACTGCTTGCACCATTTT, TTGGAGTGCTAATGGGGAAT |
| BB0532 | HP | none | GCTCAACATTGGGCCTTAAA, TTTGAACATTGAATCGTGAGAA |
| BB0539 | CHP | none | ATGGGCTTATGGGGAATCAT, GCTACAACCGCCATTCTGTT |
| BB0543 | HP | none | GTTCGGATGGAACTGCAAAT, CCATTGTTCCCAGTTCTGCT |
| BB0563 | HP | 35 | GGCAGTAAGTCAGGCTCAGG, TCCTGCGAATACTTCCCATC |
| BB0584 | CHP | 56 | TCTGCCTTTTTCATGGCTCT, CAAGTTTGATTGCGTCCTCA |
| BB0592 | HP | none | TTGCCAGAATCGGTACTTGTC, ATGGCGGTAGCTACAACAGG |
| BB0628 | CHP, LP | none | CCCAATGTCTGCCAAAGAAT, ATCCATCTCAAACCGCAGTG |
| BB0638 | nhaC-2 | 135 | TATCCGGTGCTTTTTCTGCT, ATGGAAGGGGGATGTTTACC |
| BB0671 | cheX | none | GCATGCCAAGGGTTTTGTAT, CCATCAGGCAAAGAAAAAGG |
| BB0674 | HP | none | AGGCCTACCTTTCCCAAAGA, TATTGTTTCATGGGCGACAA |
| BB0702 | kdtB | none | GCGGGTTTATTGTTGATTATGC, TTCCTTTACAAAATCCGACCTT |
| BB0708 | HP | none | GTTCGTGTTTTTGGCTGGTT, CCAGAAAGAAAAATTTTAAAAACAA |
| BB0717 | CHP | none | ATTTCCAGCGCATTTTTAGG, AAGCATATTTTTGGGCATGAA |
| BB0729 | gltP | none | TGCTGCAACCATACCCATTA, GTTCATTGCTGAGAGCACCA |
| BB0746 | oppC-2 | 41 | TGGAACAATCATTGGCATGT, TTTGCTTGCTCCCATAGCTT |
| BB0753 | MSP | none | TGTTGGTACTGCCAGCACTT, ATCAAATTCGCCCATGAAAA |
| BB0759 | HP | none | ATAATCCCAGGGGTTTCTGG, TTGAGGTTAACATTCCTGTTGC |
| BB0766 | colicin V production | none | GCCTGAGATTGCCTCAAGAC, GTGCATTAAAGGGGCGTAAA |
| BB0785 | sporulation protein G | none | GCATATGTTGCAGTTACTTTTGA, TCAAGATCGGCTGGATTTTC |
| BB0786 | ctc | none | GGGACGTCGACAAGTGGTAA, CCTTACCTTGCCCGTAAACA |
| BB0806 | HP, LP | none | CATTAGGGGTTTTGCGGATT, TCAGCATTACCGGGAACATT |
| BB0807 | CHP | none | CCACTTGGATCTATTGGCAGA, CAAAACCCTTAAGCCCAGTG |
| BB0816 | HP | none | TGCTCATTGCAGAGAAATGC, CCTGCCTGGTTGAAACATCT |
| BB0824 | HP | none | CCAGAGCAGATCATGGGATT, CCCAATTAATGGGAAAAGCA |
| BB0843 | CHP | 32 | TTGGTGGATGCAAGAAATGA, AACCTTTCACAAACGCATCC |
| BBA74 | oms28 | 171 | GCTGTTTCTGTTGCTGGTGA, ATCTCTTGCGCCTTGAGCTA |
| BBA76 | thy1 | 65 | CATTGGAGCAGGTGGTTTTT, TTCCTCTCTTGCCAAGCTGT |
| BBB04 | celB | none | ACCTTACGGTGGAGATGCTG, TTTAAAACAGCAGGCGGAAC |
| BBB09 | HP, LP | none | CGCTCCCTCTGAAACTTACG, TCACAGGAGGCTCCATTTTT |
| BBB18 | guaA | none | GGCCTACAATTCCATCCAGA, CTGTGCCACCAGAAAGTCCT |
| BBB22 | HP | 94 | CAGTGGCAGCAAAAGGAAAT, TGTATGCGGTTACGGTTGAA |
| BBB23 | CHP | 94 | CAACAAAAGGTGGCATGTTG, TTTTCCACCCTCAGCAATTC |
| BBD10 | HP, LP | none | TTTGAGGCTAAAGGAGAGTTGG, CATTTCCCAAGGCAACAAAT |
| BBD15 | HP, family 85, LP | 85 | CAAGATAGATGGGGCTTCTCA, TTTGAAACATTAGATACTCTGGAAGAA |
| BBE08 | HP, LP | none | TTATTTTTGTGTTGATAAGTTCTTGC, GCATAAAAATACCTTTGCTTCTTTTT |
| BBE19 | plasmid partition | 32 | AAAGGAGGTGTTGGCAAAAG, GGTTGATGCTTGTGGATCG |
| BBG02 | CHP, LP | 102 | CACTTGGCAAACTTGGAACA, AGGGCATTTCTCTGCCTTTT |
| BBH06 | HP | none | TGCCAGACATGTTGCTGATT, CCCCCTCAAGTTCTACAGCA |
| BBI26 | drug transport | 105 | GGACTTTGCCACATGCTGTA, GTCAATCGTGCCAGTGCTAA |
| BBI31 | CHP | 48 | TATATTGGTGGTGGCGCAGT, GTGAGGCTATGTGGGATGCT |
| BBJ09 | OspD, LP | none | AATGAAGGCGCAAATTCAAA, TTGCCTCATTTGATGCATTT |
| BBK48 | P37, LP | 75 | CGCCGATCAGGTTATAGACAA, CATATCGGGTTGCATGTCTG |
| BBL39 | ErpA8, LP | 162 | GGGACATTCGGCTACATTCT, CCCCCGCACTGTTATTAATTT |
| BBM27/P27 | Rev, LP | 63 | AAATGATTCTTCTGGAGGCAAA, TTGGCCGCTAATTTATCCTG |
| BBN38 | ErpA, LP | 162 | GTTTAAACGCTGGGGGACAT, ACGCAATATGTTCAGCACCA |
| BBN39 | erpB2, LP | 163 | GTGCTGTTTTTGCGCTGATA, TCGCCCTGCATTAATTCTTC |
| BBO39 | ErpL, LP | 164 | GCAAAGAAAAAGGGGGAGAG, TCACTGCCGCCATTAGAATA |
| BBO40 | ErpM, LP | 163 | GCAGGGCGATGATCCTAATA, GCTTTTGACTTTGCTTCTCCA |
| BBP38 | ErpA, LP | 162 | TGATGAGCAAAGCAATGGAG, GAATGTCCCCCAGCGTTTA |
| BBQ05 | antigen, P35, LP | 60 | GACACCCCGCAATCTAAAAA, AAATGCCTCCGAATCTGTTG |
| BBQ46 | HP | none | GATTAAAAATGTAATTTATATTTTACC, CTATCCTACAATCCAAATTTTG |
| BBQ47 | ErpX, LP | 163 | GCAAGATTGATGCAACTGGT, TTTTTGCCAATTCATCTGCT |
| BBS41 | OspG, LP | 164 | CTTGCAAGATTGATGCGAGT, GGGTGTGTTATCGTGGGAAC |
| BBS42 | bapA | 95 | TTGGACGCTCTTGAGGCTAT, GCAAATCAGCCAAAATTTGTT |

Abbreviations: CHP, conserved hypothetical protein; HP, hypothetical protein; LP, lipoprotein; IMP, inner membrane protein; MSP, membrane-spanning protein. Designations of the open reading frame (ORF), gene function, symbol and paralog family are according to the annotations in the database (www.tigr.org).
