# Supplementary material for: A Chromosomally Encoded Virulence Factor Protects the Lyme Disease Pathogen against Host-Adaptive Immunity
Source: PLoS Pathog. 2009 Mar 6;5(3):e1000326. doi: 10.1371/journal.ppat.1000326 (PMC2644780; doi:10.1371/journal.ppat.1000326)
Supplement: Table S2 — Oligonucleotide primers used in the study (0.07 MB DOC) [file ppat.1000326.s006.doc]

| **Sequence (5’ to 3’)** | **Purpose** |
| --- | --- |
| TTGCTGATCAAGCTCAATATAACCA | Forward primer for *B. burgdorferi flaB* Quantitative RT-PCR (qRT-PCR). |
| TTGAGACCCTGAAAGTGATGC | Reverse primer for *B. burgdorferi* *flaB* qRT-PCR. |
| AGAGGGAAATCGTGCGTGAC | Forward primer for mouse *β-actin* qRT-PCR. |
| CAATAGTGATGACCTGGCCGT | Reverse primer for mouse *β-actin* qRT-PCR. |
| GAAATTGCCAACAGTAGTCC | Forward primer for *lmp1* qRT-PCR or RT-PCR. |
| GGTCTTCTTCTTTTGGGTTT | Reverse primer for *lmp1* qRT-PCR or RT-PCR. |
| GGGGTGAAATCGACTACATA | Forward primer for *bb0209* qRT-PCR or RT-PCR. |
| GAAGTTATTTTGCCCTGTTG | Reverse primer for *bb0209* qRT-PCR or RT-PCR. |
| AGAAATGACAAACCAACCAG | Forward primer for *bb0211* qRT-PCR or RT-PCR. |
| AATGTTTTCCCTGAGTGTTG | Reverse primer for *bb0211* qRT-PCR or RT-PCR. |
| GG*TCTAGA*TTTTAGCGATTTCACACAAT | Primer P1, forward primer used to amplify the 5' flanking region to generate the *lmp1* mutant. The italicized *XbaI* site is included for cloning. |
| AA*GGATCC*TTATTCATCTATGTATAACTCC | Primer P2, reverse primer used to amplify the 5' flanking region to generate the *lmp1* mutant. The italicized *BamHI* site is included for cloning. |
| GA*CTCGAG*TAAAACCCATTGATCTTGAA | Primer P3, forward primer used to amplify the 3' flanking region to generate the *lmp1* mutant. The italicized *XhoI* site is included for cloning. |
| GA*GGTACC*TTCTTCCGATGATATTTTTG | Primer P4, reverse primer used to amplify and clone the 3' flanking region to generate the *lmp1* mutant. The italicized *KpnI* site is included for cloning. |
| CAATGAAATTTTAAACTACTTACGTGC | Primer P5, forward primer upstream of *lmp1* locus used to confirm the deletion of *lmp1*. |
| GGTTGCATTCGATTCCTGTT | Primer P6, forward primer used for the detection of insertion of Kanamycin cassette. |
| ATTCCGACTCGTCCAACATC | Primer P7, reverse primer used for the detection of insertion of Kanamycin cassette. |
| TCTGACAGAAACGATGATGTT | Primer P8, forward primer in *lmp1* used to confirm the deletion of *lmp1*. |
| CTCTAGGTACTGAGGCTTTTT | Primer P9, reverse primer used to confirm the deletion of *lmp1*. |
| AATAATAAGAAAG*CATATG*AATAAAAAACATACAAATTTTTCGG | Forward primer used for complementation of *lmp1*. The italicized *NdeI* site is attached for cloning. |
| GG*GTCGAC*TTATATTCCTATTATTTTTTTAAT | Reverse primer used for complementation of *lmp1*. The italicized *SalI* site is attached for cloning. |
| GC*GGATCC*TGTCTGTCGCCTCTTG TGGCT | Forward primer for *flab* promoter. The italicized *BamHI* site is attached for cloning. |
| AATAATAAGAAAG*CATATG*TCATT CCTCCATGATAAAATTT | Reverse primer for *flab* promoter. The italicized *NdeI* site is attached for cloning. |
| GA*GGATCC*TACTATATATATCAAAGCAA | Forward primer for truncated Lmp1 protein. The italicized *BamHI* site is attached for cloning. |
| AA*CTCGAG*CTCTAGGTACTGAGGCTTTTT | Reverse primer for truncated Lmp1 protein. The italicized *XhoI* site is attached for cloning. |

**Table S2**: Oligonucleotide primers used in the study.
